# Supplementary material for: Retrospective analysis of the BariClip procedure: Clinical outcomes and complication profile
Source: PLoS One. 2025 Nov 25;20(11):e0337334. doi: 10.1371/journal.pone.0337334 (PMC12646432; doi:10.1371/journal.pone.0337334)
Supplement: S1 File — S2 Table. Mauchly’s Test for Sphericity. S3 Table. Sphericity Corrections. S4 Table. Post Hoc Pairwise Comparisons with Bonferroni. S5 Table. Sensitivity analysis of weight loss outcomes after exclusion of the patient who underwent clip removal. (ZIP) [file pone.0337334.s001.zip › Supporting Information 3.docx]

**S3 Table: Sphericity Corrections**

| Effect | GGe | DF[GG] | p[GG] | HFe | DF[HF] | p[HF] |
| --- | --- | --- | --- | --- | --- | --- |
| Timepoint | 0.301 | 1.51, 121.96 | 2.87e-26 | 0.306 | 1.53, 123.76 | 1.28e-26 |
