# Supplementary material for: Retrospective analysis of the BariClip procedure: Clinical outcomes and complication profile
Source: PLoS One. 2025 Nov 25;20(11):e0337334. doi: 10.1371/journal.pone.0337334 (PMC12646432; doi:10.1371/journal.pone.0337334)
Supplement: Attachment — Submitted filename: Response Reviewers.docx [file pone.0337334.s004.docx]

**Response to the Editor and Reviewers**

We sincerely thank the Editor and Reviewers for their thoughtful and constructive feedback on our manuscript. We have carefully revised the manuscript to address all comments and to ensure compliance with *PLOS ONE*’s formatting and reporting requirements.

Below, we provide a detailed, point-by-point response to all reviewer comments, along with corresponding revisions in the manuscript.

Reviewer #2:

| Comments | Response | Modification - Manuscript |
| --- | --- | --- |
| - Depth of Comparison While you have cited literature on LAGB and LSG, the comparison remains largely descriptive and does not include your own data beyond weight loss. Readers would benefit from clearer, data-driven contrasts on outcomes such as comorbidity resolution, GERD, and quality of life. If such data are not available in your cohort, please be explicit about this limitation and avoid overstating the conclusions. | We thank the reviewer for this important comment. As this was a retrospective study, our dataset was limited to weight loss and perioperative outcomes. Systematic documentation of comorbidity resolution, GERD incidence, and quality-of-life measures was not consistently available in the medical records and therefore could not be analyzed with sufficient accuracy. We have revised both the Discussion and Limitations sections to explicitly acknowledge this limitation and to clarify that our comparisons with LAGB and LSG regarding these outcomes are based on published evidence rather than our own data. We have also ensured that our conclusions are appropriately tempered to reflect this limitation. | *Discussion and limitation: line: 292-295*  *“These findings suggest that BariClip may offer advantages in operative efficiency, early recovery, and perioperative safety, although our dataset was limited to weight loss and perioperative outcomes. Systematic data on comorbidity resolution, GERD incidence, and quality-of-life measures were not available in this retrospective cohort, which represents a limitation of our study.”*  *Line: 364-368*  *“Additionally, detailed documentation of comorbidity improvement (e.g., type 2 diabetes, hypertension, dyslipidemia, and obstructive sleep apnea) was not routinely available in the patient records; therefore, our analysis was restricted to weight loss and perioperative parameters. We acknowledge this as a limitation and emphasize that future prospective studies are needed to evaluate these clinically important outcomes”* |
| - Comorbidities and GERD The absence of systematic comorbidity and GERD outcomes is a significant weakness. If you cannot provide reliable retrospective data, please present this candidly as a limitation and strengthen your discussion with relevant published evidence. | We agree with the reviewer. Systematic and reliable comorbidity and GERD outcomes were not available retrospectively in our patient records, which was explicitly in the Limitations section.  To address this gap, we have expanded the Discussion by including recent evidence from the literature regarding the effect of bariatric procedures on comorbidities and GERD, highlighting where BariClip may be expected to perform similarly or differently based on its mechanism of action. | Discussion:  *line: 297-306*  *“Nevertheless, insights from the broader bariatric literature provide useful context. LSG has consistently demonstrated substantial improvement in obesity-related comorbidities such as type 2 diabetes, hypertension, and dyslipidemia, though it carries an increased risk of de novo or worsened GERD (18,19). LAGB generally achieves more modest comorbidity resolution and is burdened by late device-related complications such as slippage, erosion, and reoperation (20). Early BariClip data from Bonaldi et al. similarly reported promising short-term weight loss but noted a non-negligible incidence of GERD symptoms during follow-up (21). Future prospective studies with standardized collection of comorbidity and GERD outcomes will be essential to establish whether BariClip* *offers a more favorable risk–benefit profile compared with LAGB and LSG.”* |
| - Positioning of BariClip The section on future directions is clearer, but the clinical role of BariClip still needs sharper definition. Is it primarily an alternative to Lap-BAND, a competitor to ESG, or a bridge procedure? Please expand this discussion to help readers understand its potential place in practice. | We thank the reviewer for this insightful comment. We agree that clarifying the clinical role of BariClip is important. In the revised Discussion, we have added a section that positions BariClip relative to other restrictive procedures, emphasizing its potential as an alternative to LAGB, its overlap with ESG, and its possible role as a bridge procedure in selected patients. | Discussion  Line: 350-358  *“ In this context, BariClip can be positioned in several ways: as an* ***alternative to laparoscopic adjustable gastric banding (LAGB)****, given its reversibility without the long-term device-related complications commonly reported with bands; as a potential* ***competitor to endoscopic sleeve gastroplasty (ESG)****, offering a surgical yet anatomy-preserving restrictive option that may provide greater durability than endoscopic approaches; and as a* ***bridge procedure*** *for high-risk or super-obese patients, where initial weight loss can reduce operative risk and enable later conversion to a more definitive resectional bariatric surgery if required (27). Future comparative studies are needed to clarify which of these roles will be most clinically relevant.”* |
| - Manuscript Format Given the limitations of the dataset, you might consider presenting this as a short communication or technical report. This would better align expectations with the scope of your data and highlight the study as an early contribution to the growing evidence base for BariClip, rather than a definitive outcomes study. | We thank the reviewer for this perspective. However, we respectfully believe that the current format is appropriate for the manuscript. As clearly stated in both the Methods and Discussion sections, this study focuses on **short-term outcomes** of the BariClip procedure. Similar early outcome studies on new bariatric procedures have also been presented as full original articles in the literature, and we believe maintaining this format allows for a more comprehensive presentation of perioperative details, weight loss outcomes, and context within the existing evidence base. We have further clarified in the Discussion that our findings represent **short-term results** and acknowledge the need for future studies with longer follow-up and broader outcome measures. | *NA* |
